# Biology Teachers’ Worldviews on the Global Distribution and Loss of Biodiversity: A GIS-Based Mental-Mapping Approach

**DOI:** 10.3389/fpsyg.2019.01021

**Published:** 2019-05-14

**Authors:** Florian Fiebelkorn, Susanne Menzel

**Affiliations:** Didactics of Biology, Faculty of Biology and Chemistry, Osnabrück University, Osnabrück, Germany

**Keywords:** student teachers, education for sustainable development, biodiversity, mental maps, spatial optimism bias, overestimation bias

## Abstract

This paper explores (1) student teachers’ mental maps of the global distribution and loss of biodiversity and (2) their perception of threatened biodiversity at the national, transnational and global levels. Data was collected from a questionnaire study of student biology teachers from Germany (*n* = 868) and Costa Rica (*n* = 284). Student teachers’ mental maps matched quite well with the scientific view. Nevertheless, they clearly showed a “brazilisation bias,” meaning that the first and foremost country associated with high and threatened biodiversity was Brazil. Industrialized countries were often misconceived to have a particularly threatened biodiversity. Except for Brazil (and Costa Rica in the Costa Rican sample), most students neglected a connection between a country’s high biodiversity and its high threat as proposed by the biodiversity hotspots concept. Despite this common ground, major ethnocentric distortions merged in the composite mental maps for each sample: German students had a more global perspective on biodiversity and its loss, whereas Costa Ricans students had a more localized view. Student teachers from both countries have largely overestimated the percentage of threatened plant species on a national, transnational and global level (“overestimation bias”). In addition, the estimated percentage of threatened plant species have correspondingly increased with a greater distance from the students’ home country (“spatial optimism bias”). Results will be discussed in terms of educational implications.

## Introduction

The loss of biodiversity progresses on a global scale and is considered one of the most serious environmental problems of our time (Dirzo and Raven, 2003; Millennium Ecosystem Assessment [MEA], 2005). Education for Sustainable Development (ESD) is one important counteraction to this trend by making the public aware of the role and value of biodiversity and the steps needed to conserve it (United Nations Conference on Environment and Development [UNCED], 1992; Secretariat of the Convention on Biological Diversity, 2010). Within the framework of ESD, the distribution and loss of biodiversity has a high potential to be used a model context for discussing global challenges and conservation strategies in the science classroom (Gayford, 2000; Kassas, 2002; Scheunpflug and Asbrand, 2006; Menzel and Bögeholz, 2008; Lindemann-Matthies et al., 2011). Worldwide school systems provide the largest organized base for such a biodiversity education (Nagra, 2010).

In the past, traditional environmental education was merely focused on local contexts and ecological facts, whereas global cause–effect relationships were largely marginalized (Bolscho and Hauenschild, 2006). In contrast, ESD should be interdisciplinary and global in its scope (Scott and Gough, 2004; Summers et al., 2005; Scheunpflug and Asbrand, 2006; Menzel and Bögeholz, 2008). This is a difficult task as the scientific evidence about global biodiversity and its loss is rather uncertain and often controversial (Kassas, 2002). To adequately teach this complex issue, teachers need a basic understanding about the world, its principal regions and political and biogeographic characteristics (Holm and Farber, 2002). In line with other authors, we argue that teaching about a complex and controversial issue, such as the distribution and loss of biodiversity, raises important questions for teachers concerning bias, balance and personal worldviews (e.g., Pajares, 1992; Holm and Farber, 2002; Moseley and Utley, 2008).

To date, researchers have been predominantly focused on student teachers’ understanding of the terminology and socio-scientific aspects of biodiversity issues (Gayford, 2000; Summers et al., 2004; Kyburz-Graber et al., 2006; Lindemann-Matthies et al., 2011). Besides these important aspects, the global dimension of the distribution and loss of biodiversity is often depicted as a major educational challenge for both teachers and learners alike (Bybee, 1991; Chiodo, 1993; Merryfield, 2000; Hicks and Bord, 2001; Holm and Farber, 2002; Bolscho and Hauenschild, 2006; Scheunpflug and Asbrand, 2006; Menzel and Bögeholz, 2008; Lindemann-Matthies et al., 2009). With regards to the student teachers’ worldviews and perceptions of the global dimension of biodiversity and its loss, a sound empirical basis is still lacking. How student biology teachers from Germany and Costa Rica perceive the global distribution and loss of biodiversity was investigated in this study. To achieve an effective understanding of this, we explored (1) student teachers’ mental maps of the global distribution and loss of biodiversity and (2) student teachers’ perceptions of threatened biodiversity at a national, transnational, and global spatial level.

## Theoretical Background

### Global Distribution and Loss of Biodiversity

In general, most of the terrestrial biodiversity can be found in tropical ecosystems, especially in the tropical rainforests of the Americas, Africa, and Southeast Asia (Mittermeier et al., 2004). High diversity is also found in temperate regions with a Mediterranean climate, e.g., Southwest Australia, the Cape region of South Africa, California, central Chile, and the Mediterranean basin (Groombridge and Jenkins, 2002; Mutke and Barthlott, 2005; Primack, 2010).

In a strict scientific sense, biodiversity is defined as “the variability among living organisms from all sources, including inter alia, terrestrial, marine and other aquatic ecosystems and the ecological complexes of which they are part: this includes diversity within species, between species and of ecosystems” (United Nations Conference on Environment and Development [UNCED], 1992; p. 3). Data on global plant diversity is assumed to serve as one of the best surrogates for the total diversity of the living creatures found on our planet (Dirzo and Raven, 2003; Mutke and Barthlott, 2005). Moreover, on a global level, estimates for plant diversity are much more precise than those pertaining to animal diversity (Groombridge and Jenkins, 2002; Mutke and Barthlott, 2005; Primack, 2010). Thus, for the purpose of this paper, we used “plant diversity” as an indicator for “biodiversity.”

In total, more than 270,000 species of plants have been described scientifically (Walter and Gillett, 1998). The top ten countries that hold the highest biodiversity in terms of number of plant species are: Brazil (56,215), Colombia (51,220), China (32,200), Indonesia (29,375), Mexico (26,071), South Africa (23,420), Venezuela (21,073), United States (19,473), Ecuador (19,362), and India (18,664) (in parenthesis number of total plant species) (Groombridge and Jenkins, 2002).

The Red List of Threatened Species^TM^ (RL), which is compiled by the International Union for Conservation of Nature (IUCN), is “widely recognized as the most objective and authoritative listing of species that are globally at risk of extinction” (IUCN, 2004, p. Xi). According to the RL, the following countries hold the largest number of threatened plant species: Ecuador (1,837), Malaysia (694), China (452), Indonesia (394), Brazil (387), Cameroon (378), India (314), Tanzania (298), Sri Lanka (285), and Madagascar (280) (in parenthesis number of total threatened plant species) (IUCN, 2011). Globally, more than 4.3% of all plant species have been classified as being threatened (IUCN, 2011).

Earth’s richest and simultaneously most threatened reservoirs of biodiversity are so-called biodiversity hotspots (Myers et al., 2000). Many biodiversity hotspots are found in the developing countries of the tropics, including Costa Rica. Scientifically speaking, a region has to meet two strict criteria to be designated as a biodiversity hotspot: (1) it has to contain at least 1,500 endemic plant species and (2) 70% of its pristine vegetation has to be destroyed (Myers et al., 2000). On a global scale, the concept of biodiversity hotspots is one of the most established biodiversity conservation templates (Mittermeier et al., 2004).

Costa Rica and Germany are regarded as international role models for the successful implementation of ESD on all educational levels (Guier et al., 2002; Seybold and Rieß, 2006; Blum, 2008). Both countries acknowledged ESD as an important component of their National Biodiversity Strategy and Action Plans (NBSAP’s) (Ministerio del Ambiente y Energía [MINAE], 2000; Küchler-Krischun and Walter, 2007). Furthermore, the global dimension of the distribution and loss of biodiversity is an integral part of their national secondary science curriculums (Ministerio de Educación Pública [MEP], 2003, 2005; Sekretariat der Ständigen Konferenz der Kultusminister der Länder in der Bundesrepublik Deutschland [KMK], 2004). Thus, Costa Rican and German secondary biology teachers are required to incorporate biodiversity-relevant topics into their secondary classrooms, including its global dimension (Ministerio de Educación Pública [MEP], 2003, 2005; Sekretariat der Ständigen Konferenz der Kultusminister der Länder in der Bundesrepublik Deutschland [KMK], 2004).

### A Constructivist View on Student Teachers’ Mental Maps

Despite their future role as teachers, in this study we viewed student teachers as learners. We defined “learning” as the active and individual construction of knowledge (Piaget, 1971; de Kock et al., 2004) which is shaped and filtered through social interactions and cultural experiences (Vygotskij, 1978; Windschitl, 2002; Bless et al., 2004). Based on this constructivist model of learning, we assumed that student science teachers from Costa Rica and Germany would hold an individually built knowledge toward the global distribution and loss of biodiversity which is partly shaped by their different social and cultural settings.

According to Bell (2009) these “internal spatial representation of the world” constitute our “cognitive maps.” Thus, “cognitive maps” exist only in the mind of people and are influenced by social interactions and cultural experiences. The term “mental map” is often attached to different meanings across different disciplines including environmental psychology, anthropology, cognitive science, and human geography (Kitchin, 2002; Bell, 2009). For the purpose of this paper we defined a “mental map” as an external map-like product that represents the worldviews of individuals or groups pertaining to the spatial and environmental relations of geographic space (Kitchin, 2002; Bell, 2009). In geographical and educational research, mental maps can offer a promising way to illustrate and analyze individuals’ and groups’ geographic literacy and worldviews (e.g., Chiodo, 1993; Saarinen and MacCabe, 1995; Pinheiro, 1998; Chokor, 2003; Saarinen, 2005). As many cultural sources and factors underlie our mental maps of the world, ethnocentric deviations and distortions are likely to occur when they are compared to reality (Saarinen and MacCabe, 1995; Pinheiro, 1998; Saarinen, 2005). Some authors argue that such differences in mental maps can account for many environmental conflicts in our world (e.g., Koger and Du Nann Winter, 2010). In the present study, composite mental maps were used to represent secondary pre-service teachers’ worldviews on the distribution and loss of biodiversity. To the authors’ knowledge, this study is the first to attempt an assessment of student teachers’ biogeographic worldviews regarding the distribution and loss of global biodiversity through the use of mental maps.

### Perceived Threat of National, Transnational, and Global Biodiversity

#### Spatial Optimism Bias

Recent research in environmental psychology revealed that people are generally more concerned about global environmental problems than about national ones (Dunlap and Mertig, 1995; Uzzell, 2000; Gifford et al., 2009). Gifford et al. (2009) demonstrated in a multinational survey with the general public that there seems to exist a so-called “spatial optimism bias” when evaluating environmental problems from the national to the global scale. It was found that assessed environmental problems increased as the spatial level increased from the national to the global level, regardless of whether the subjects were from a developed or a developing country (Gifford et al., 2009).

#### Overestimation Bias

People seem to have only very vague ideas about the current number of animal and plant species, as well as about respective extinction rates on the national and on the global level (Dunning, 1997). Lindemann-Matthies and Bose (2008) found the general public of Switzerland to drastically overestimate the overall amount of Swiss and global plant species. Dunning (1997) found the same trends when asking United States undergraduates about the total number of species on earth and the number of species going extinct each year. This phenomenon, which we will henceforth refer to as “overestimation bias,” may have serious consequences in conservational and educational terms: Convincing people that biodiversity loss is a serious problem becomes more difficult when people’s perception of the current situation is so different from reality (Dunning, 1997; Lindemann-Matthies and Bose, 2008). Moreover, scientifically correct numbers of threatened species may appear quite low to people who have much higher numbers in mind. As a consequence, reality may be perceived as better than it really is.

## Research Questions and Aim of the Study

Our study was guided by the following research questions: (1) To what degree do biology teachers from an industrialized country and a biodiversity hotspot share a common view on Earth’s biodiversity? (2) And in what ways do they differ? More in detail we aimed at exploring student teachers’ mental maps of the global distribution and loss of biodiversity and whether their perceived threat of biodiversity at the national, transnational and global spatial levels will be affected by a “spatial optimism bias” and an “overestimation bias.”

With regard to the first aim of the study we assumed that distinctive ethnocentric perspectives, such as living at a biodiversity hotspot (Costa Rica) and living in an industrialized country (Germany) would be likely to merge on aggregated mental maps when compared to scientific data. As the loss of biodiversity becomes especially apparent in biodiversity hotspots (Myers et al., 2000), we were particularly interested in whether student teachers from both countries will hold a naive concept of biodiversity hotspot, meaning that countries of assumed high plant diversity will also be suspected of having a high amount of threatened plant species. As Costa Rica forms part of the Mesoamerica biodiversity hotspot, we expected that Costa Rican students would be more likely to hold a biodiversity hotspot concept than German students.

Regarding our second aim, based on the literature cited above, we hypothesized that: (i) student teachers from both countries will generally overestimate the percentage of threatened plant species on a national, transnational and global level when compared to scientific data; (ii) participants from both countries will perceive the percentage of threatened plant species on a global level as more serious than at the transnational level, and this, in turn, higher than at the national level; and (iii) students from both countries will perceive the threat of plant species in their own country as less severe than in the respective other country.

## Materials and Methods

### Sample

For the present study we carried out a quantitative questionnaire survey in Winter 2010/2011 with secondary pre-service science teachers in Germany (*n* = 868; *M*_age_ = 23.1, *SD* = 3.3; female: 75.2%) and Costa Rica (*n* = 284, *M*_age_ = 25.8, *SD* = 6.6; female: 55.3%). All Costa Rican participants were secondary natural science (= biology, chemistry, and physics) teachers and all German participants were secondary biology teachers. The German sample comprised of students from 23 different public universities. In Germany participants per university varied between 6 (Berlin – Freie Universität) and 105 students (Osnabrück). In Costa Rica, students from three public and three private universities participated in the study. Costa Rican participants per university varied between 16 (Universidad Americana) and 72 students (Universidad Florencio del Castillo). Within the German sample 46.3% of the students were at the beginning of their studies (≤ 4 terms) and 53.3% were advanced students (> 4 terms) (0.3% no answer). In the Costa Rican sample we found that 37.7% were beginning students and 47.2% advanced students (15.1% no answer). A detailed description of the sample can be found in Table 1. The gross enrollment ratio in tertiary education in 2015 is comparable in both countries and was 53.6% in Costa Rica, and 66.3% in Germany (OECD, 2018). Despite the fact that there are good scholarship programs for students, the education systems in both countries still show a strong socio-economical selectivity. School leavers with low socio-economic status are less likely to enter higher education than young people with high socio-economic status (Berthold and Leichsenring, 2012; CONARE, 2015; Autorengruppe Bildungsberichterstattung, 2018).

**Table 1:** Characteristics of the pre-service biology^1^ teacher sample from Germany (*n* = 868) and Costa Rica (*n* = 284).

Nationality	University	Total^2^	BS	AS	Females [%]
Germany	Berlin (Freie Universität)	6	3	3	100
	Berlin (Humboldt Universität)	26	13	12	69.2
	Bielefeld	54	5	49	77.8
	Braunschweig	27	27	0	88.9
	Bremen	26	19	6	73.1
	Dortmund	23	0	23	87.0
	Duisburg-Essen	21	0	21	76.2
	Erlangen-Nürnberg	59	31	28	74.6
	Halle-Wittenberg	21	9	12	71.4
	Hamburg	70	32	38	75.7
	Hannover	24	5	18	70.8
	Jena	56	53	3	75.0
	Karlsruhe^3^	32	1	31	84.4
	Köln	12	5	7	66.7
	Leipzig	26	0	26	65.4
	Marburg	72	48	24	70.8
	München	18	9	9	83.3
	Münster	38	14	24	71.1
	Oldenburg	39	23	16	76.9
	Osnabrück	105	48	57	78.1
	Potsdam	24	1	23	79.2
	Rostock	59	52	7	71.2
	Vechta	30	4	26	63.3
Costa Rica	Universidad Americana (UAM)^4^	16	10	3	37.5
	Universidad de Costa Rica (UCR)	60	20	40	51.7
	Universidad Estatal a Distancia (UNED)	41	5	34	61.0
	Universidad Florencio del Castillo (UCA)^4^	72	35	27	51.4
	Universidad Nacional (UNA)	67	37	30	55.2
	Universidad de San José (USJ)^4^	28	21	6	75.0

*BS, Beginning students (≤ 4 terms of study); AS, Advanced students (> 4 terms of study). ^1^All German participants were pre-service biology teachers, whereas all the Costa Rican participants were pre-service natural science teachers (= biology, chemistry and physics). ^2^Deviations in the total of students with BS and AS values can be attributed to the fact that not all students have indicated their terms of study. ^3^University of Education (= “Pädagogische Hochschule”). ^4^Private universities. The higher education system in Costa Rica is dominated by five public universities, of which only the Universidad de Costa Rica (UCR), Universidad Nacional de Costa Rica (UNA) and the Universidad Estatal a Distancia (UNED) offer natural sciences teacher education programs. In addition, there are more than 59 private universities with different quality and, in some cases, relatively high university fees. Of those private universities, only a few offer teacher education programs in the natural sciences (CONARE, 2015; DAAD, 2018). In Germany, teacher education is offered exclusively at state universities. In Germany there are a total of 87 public universities (including the seven Universities of Education in Baden-Württemberg; “Pädagogische Hochschule”), of which 51 offer biology teacher education programs (https://hochschulkompass.de).*

### Data Collection

For data collection the persons in charge of tertiary science teacher education in both countries were contacted and asked for participation within the project. In Germany, all questionnaires were sent to the respective persons in charge accompanied by a standardized information sheet on how to conduct the questionnaire survey. In Costa Rica the corresponding author of the study conducted all questionnaire surveys on-site with the help of local collaborators. In both countries, the questionnaires were administered in a paper-and-pencil format. Prior to the completion the questionnaires, students were informed that the survey was about their ideas and opinions regarding biodiversity. On the first page of the questionnaire, students were given a definition of biodiversity, which was based on the definition of the CBD (United Nations Conference on Environment and Development [UNCED], 1992). To avoid bias, participants in both countries were not informed that they were taking part in an intercultural study until they completed the questionnaire. The questionnaires were presented in the respective mother tongue, Spanish in Costa Rica and German in Germany. Data collection took place in class sets in each university. The questionnaires were filled out by the students individually under exam-like conditions. The time for the completion of the herewith presented measures took about 5 min.

### Measures

#### Socio-Demographic Variables

To gather basic information about our participants, we collected socio-demographic variables such as nationality, attended university, age, sex, and current semester.

#### Mental Maps of Global Biodiversity

The process of assessing individuals’ or groups’ concepts about spatial and environmental relations of geographic space with the final objective of generating a map representation is called “mental mapping” (Bell, 2009). In this study we followed an indirect mental mapping approach by asking the participants to name three countries with a particularly high plant diversity and three countries with a particularly threatened plant diversity. The original survey wording was: “Please name three countries that you think have a particularly high diversity of plant species.” and “Please name three countries where you think the diversity of plant species is particularly threatened.”

#### Perceived Threat of Biodiversity

In order to evaluate student teachers’ perception of threatened biodiversity on the national, transnational and global spatial levels, they were asked to estimate the percentage of threatened plant species in Germany, Costa Rica, and worldwide. Original survey wording in the German questionnaire was: “Please estimate what percentage of flowering plants are threatened in Germany, Costa Rica, and worldwide.” In the Costa Rican version of the questionnaire, the question was phrased as: “Please estimate what percentage of flowering plants are threatened in Costa Rica, Germany, and worldwide.” In both Germany and Costa Rica, the question was first asked with regards to the respondents’ own country, followed by the other country and finally about the percentage of threatened plants in the world. For the answering of the question, there were ready-made spaces to enter the percentages (e.g., “Germany _____ %”).

### Analysis

#### Mental Map Production

Firstly, all mentioned country names of assumed high and/or threatened plant diversity were standardized, for example notions such as “United States,” “United States of America,” “US,” or “USA” where all coded as “USA.” Responses that could not clearly be assigned to a specific country were excluded from analysis (e.g., Andes, hot regions etc.). Hereafter, we formed a variable for each of the reported countries. The variables (= countries) were coded as “1” when a participant assumed a country to have a particularly high plant diversity, as “2” when a country was assumed to have a particularly threatened plant diversity, and as “3” when a country was considered to have a particularly high and threatened biodiversity – thus, resembling the concept of biodiversity hotspots. Frequencies of notions for each country and category were calculated using SPSS. Tabular frequency data of students’ notions and the scientific data of the total number and the number of threatened plant species per country were exported to ArcGIS^TM^ (GIS; Geographical Information Systems), a software infrastructure for the production of geographical information and maps. When exporting, the frequency data from SPSS is linked with the respective geo-referenced countries within an ArcGIS^TM^ database, which can then be visualized via ArcMap^TM^ (a module of the ArcGIS software) in a world map, complete with different shadings for countries that have a high or threatened biodiversity. Respective scientific data for total plant species counts per country were taken from Groombridge and Jenkins (2002) and for threatened plant species from IUCN (2011).

**FIGURE 1:**
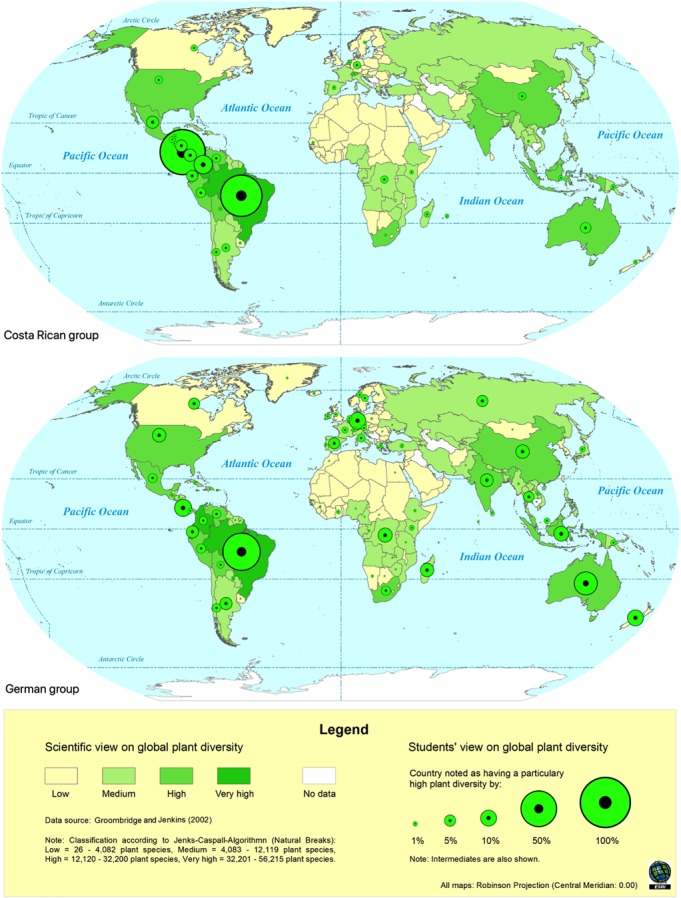
Composite mental maps of countries assumed to have a high plant diversity of Costa Rican (top) and German (bottom) student biology teachers. Within each map the scientific view on global plant diversity (green shading) is overlaid with the respective student teachers’ view (green circles).

**FIGURE 2:**
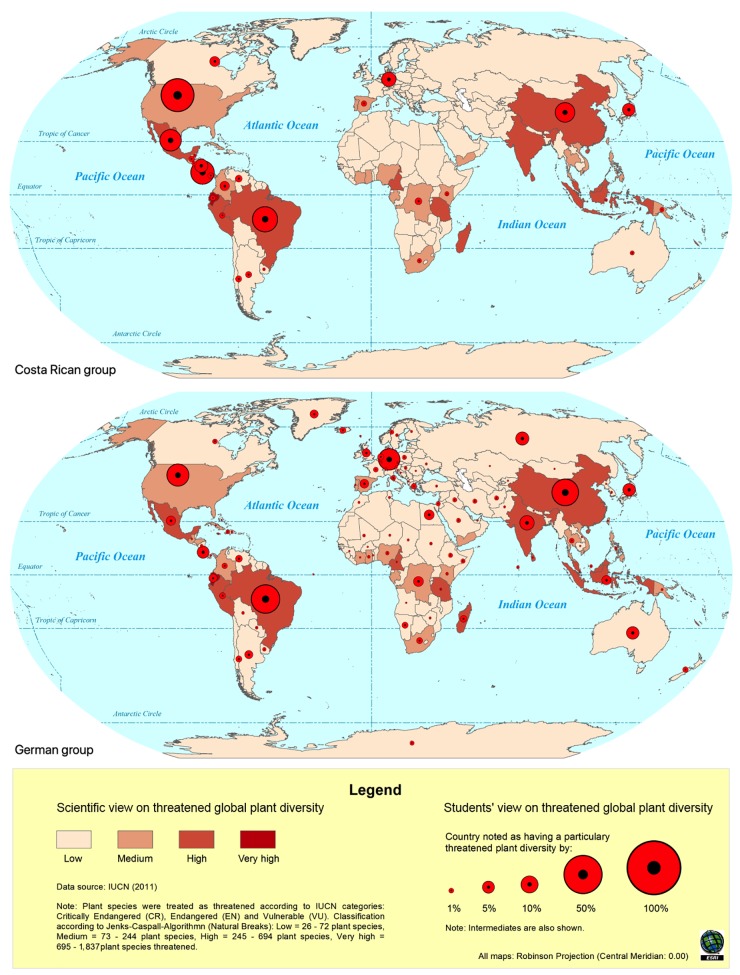
Composite mental maps of countries assumed to have a threatened plant diversity of Costa Rican (top) and German (bottom) student biology teachers. Within each map the scientific view on threatened global plant diversity (red shading) is overlaid with the respective student teachers’ view (red circles).

**FIGURE 3:**
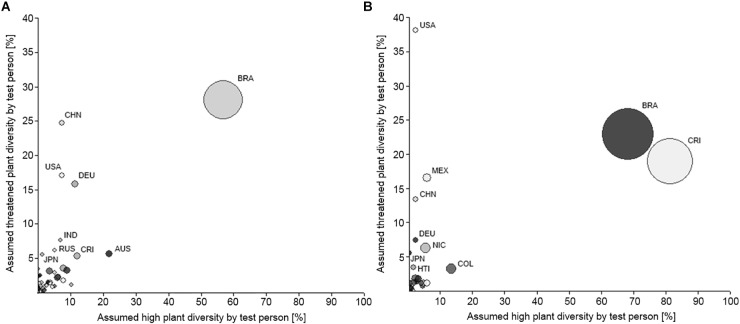
Relationships between notions of countries with an assumed high and threatened biodiversity as mentioned by student teachers from Germany **(A)** and Costa Rica **(B)**. Bubble size indicates percentage of individuals naming a certain country as having a high and threatened biodiversity. AUS, Australia; BRA, Brazil; CHN, China; COL, Colombia; CRI, Costa Rica; DEU, Germany; HTI, Haiti; IND, India; JPN, Japan; NIC, Nicaragua; RUS, Russia; USA, United States.

With the help of ArcMap^TM^ composite political world maps of student teachers’ worldviews on the distribution and threat of global plant diversity in comparison to the scientific views were created. For the map construction we overlaid the scientific views on the distribution and threat of global plant diversity with respective student teachers’ views. The resulting map patterns are presented here to illustrate the special features of each sample and characteristics common to both (see Figure 1, 2). In order to assess countries which were thought to have a particularly high and threatened plant diversity (= naive biodiversity hotspots) we created bubble charts (see Figure 3). The percentages of persons holding a naive biodiversity hotspot concept of a respective country are indicated by the size of the bubbles. All geo-spatial analysis, bubble charts and mental maps were created using ArcMap^TM^. The programs GRASS (Geographical Resources Analysis Support System^1^) and QGIS^2^ are free, readily available software on the Internet that have a similar functionality to ArcGIS^TM^.

#### Statistical Analysis

National differences between student teachers’ notions of countries with high and/or threatened plant diversity were analyzed by using chi^2^-tests. To test whether nationality was significantly related to student teachers’ estimates of the percentages of threatened plant diversity on the national, transnational and global spatial level, we used an Analysis of Variance (One-way ANOVA).

## Results

### Student Teachers’ Mental Maps of Global Biodiversity

Special features and common characteristics of each subsample’s composite mental maps on global plant diversity are shown in Figure 1, 2.

#### Worldviews on Countries With High Plant Diversity

Common characteristics of student teachers’ mental maps from both countries were: (1) the majority of student teachers in both countries mentioned Brazil as a country with particularly high plant diversity (Germany: 56.7% and Costa Rica: 68.3%), (2) most other countries were only mentioned by a small number of participants (except for Costa Rica in the Costa Rican sample, see below), and (3) students’ views matched quite good with scientific data, except for some countries as mentioned in the German sample (e.g., Germany, New Zealand, and United States). However, some of the scientific top ten countries, such as South Africa and India, were not or only marginally considered to have high plant diversity. Special features of the Costa Rican sample were: (1) the vast majority (81.3%) of Costa Rican student teachers mentioned Costa Rica as a country with high plant diversity; (2) most Costa Rican students almost exclusively focused on Latin American countries as having high plant diversity, whereas other regions of the world, such as Africa and South-East Asia, were largely marginalized (Figure 1). Special features of the German sample were: (1) German students’ notions were distributed more evenly across the globe including South- and Central America (e.g., Brazil and Costa Rica), Africa (e.g., Congo and Madagascar), and Asia (e.g., Indonesia and China), (2) German top ten country notions also included nations such as Germany and New Zealand that are described to have a rather low plant diversity from a scientific point of view. A detailed overview of the ten most frequently mentioned countries that have a high biodiversity can be found in Table 2.

**Table 2:** Frequencies of student teachers’ notions of countries holding high plant diversity.

German sample				Costa Rican sample			
Rank	Country	Scientific rank^a^	Frequency [%]^b^	Rank	Country	Scientific rank^a^	Frequency [%]^b^
1	Brazil	1	56.7^**^	1	Costa Rica	15	81.3^***^
2	Australia	13	21.8^***^	2	Brazil	1	68.3^**^
3	Costa Rica	15	12.1^***^	3	Colombia	2	13.4^***^
4	Germany	107	11.4^***^	4	Mexico	5	6.0^***^
5	New Zealand	112	10.2^***^	5	Panama	22	6.0^***^
6	Indonesia	4	8.9^***^	6	Nicaragua	32	5.6^***^
7	Congo (COD)	19	7.8^***^	7	Ecuador	9	4.9
8	Madagascar	23	7.7^***^	8	Australia	13	4.6^***^
9	China	3	7.3^**^	9	Peru	12	4.2
10	United States	84	7.3^**^	10	Venezuela	7	3.2

*^a^After Groombridge and Jenkins (2002), ^b^Significance of Chi^2^-test between notions of the German and Costa Rican sample: ^∗∗^p < 0.01, and ^∗∗∗^p < 0.001.*

**Table 3:** Frequencies of student teachers’ notions of countries holding threatened plant diversity.

German sample				Costa Rican sample			
Rank	Country	Scientific rank^a^	Frequency [%]^b^	Rank	Country	Scientific rank^a^	Frequency [%]^b^
1	Brazil	5	28.1	1	United States	14	38.1^***^
2	China	3	24.7^***^	2	Brazil	5	22.9
3	United States	14	17.1^***^	3	Costa Rica	28	19.0^***^
4	Germany	91	15.8^***^	4	Mexico	13	16.5^***^
5	India	7	7.6^**^	5	China	3	13.4^***^
6	Russia	94	6.2^*^	6	Germany	91	7.4^***^
7	Australia	9	5.7^***^	7	Nicaragua	55	6.3^***^
8	Japan	86	5.6	8	Japan	86	5.6
9	Costa Rica	28	5.3^***^	9	Haiti	65	5.3^***^
10	Congo (COD)	7	3.6^**^	10	Canada	158	3.5^**^

*^a^After IUCN (2011), ^b^Significance of Chi^2^-test between notions of the German and Costa Rican sample: ^∗^p < 0.05, ^∗∗^p < 0.01, and ^∗∗∗^p < 0.001.*

#### Worldviews on Countries With Threatened Plant Diversity

Overall, there were more similarities between both subsamples concerning their views on countries with threatened plant diversity than for countries with high plant diversity (Figure 2). Common characteristics for our participants’ views on countries with threatened plant diversity were: (1) the majority of student teachers from both countries mentioned Brazil as a country with particularly threatened plant diversity (Germany: 28.1% and Costa Rica: 22.9%), (2) industrialized or newly developed countries such as the United States, Germany, Japan, and China were considered as countries with a particularly threatened plant diversity by many students from both subsamples. Except for some countries such as Germany, Russia, Japan, and Canada the students’ view matched quite well with scientific data. However, some of the scientific top ten countries of threatened plant species such as Malaysia, Indonesia and India were not or only marginally considered to have threatened plant diversity (Figure 2). Special features of the Costa Rican sample were: (1) the majority of Costa Rican students (38.1%) considered the United States as having a particularly threatened plant diversity (even more than Brazil), (2) Costa Rican students were mainly focused on Latin American countries as having a threatened plant diversity, except for some industrialized countries such as Germany, Japan and China. Special features of the German sample were: (1) German students’ notions of countries with threatened were distributed more evenly across the globe. A detailed overview of the ten most frequently mentioned countries that have a threatened biodiversity can be found in Table 3.

#### Biodiversity Hotspots

Students’ notions of countries with particularly high and threatened plant diversity on an individual level are shown in Figure 3.

As hypothesized, our results suggest that a naive biodiversity hotspot concept exists on an individual level in both samples. Brazil is considered as a naive biodiversity hotspot by more than two in ten students from both subsamples. Additionally, nearly two in ten Costa Rican students also considered their home country a biodiversity hotspot. All other countries were mentioned to a far lesser extent, with Costa Rican students mainly focusing on Latin American countries, largely marginalizing other parts of the world. However, all mentioned “top-ten” biodiversity hotspot countries from both subsamples were in fact scientifically defined biodiversity hotspots (except for Germany in the German sample). A detailed overview of the ten most frequently mentioned countries that have a high *and* threatened biodiversity can be found in Table 4.

**Table 4:** Frequencies of student teachers’ notions of countries holding high *and* threatened plant diversity (=biodiversity hotspots).

German sample			Costa Rican sample		
Rank	Country	Frequency [%]	Rank	Country	Frequency [%]
1	Brazil^a,b^	24.0	1	Brazil^a,b^	19.4
2	Australia^c^	2.6^**^	2	Costa Rica^f^	16.9^***^
3	Indonesia^d,e^	2.6^*^	3	Colombia^i,j^	2.1^**^
4	Costa Rica^f^	2.3^***^	4	Nicaragua^f^	2.1^***^
5	Mexico^f,g^	2.2^**^	5	Congo (COD)^h^	1.4^*^
6	Congo (COD)^h^	2.0^*^	6	Mexico^f,g^	1.4
7	Ecuador^i,j^	2.0	7	Ecuador^i,j^	0.7
8	Germany	2.0^*^	8	Guatemala^f^	0.7
9	China^k,l^	1.8	9	Panama^f^	0.7
10	United States^m^	1.7	10	Venezuela^j^	0.4

*Country is part of and/or hosts a scientifically defined biodiversity hotspot: ^a^Atlantic Forest hotspot, ^b^Cerrado hotspot, ^c^Southwest Australia hotspot, ^d^Sundaland hotspot, ^e^Wallacea hotspots, ^f^Mesoamerica hotspot, ^g^Madrean Pine-Oak Woodlands hotspot, ^h^Eastern Afromontane hotspot, ^i^Tumbes-Chocó-Magdalena hotspot, ^j^Tropical Andes hotspot, ^k^Mountains of Southwest China hotspot, ^l^Himalaya hotspot, ^m^California Floristic Province hotspot (after Mittermeier et al., 2004). Significance of Chi^2^-test between notions of the German and Costa Rican sample: ^∗^p < 0.05, ^∗∗^p < 0.01, and ^∗∗∗^p < 0.001.*

#### Perceived Threat of National, Transnational, and Global Biodiversity

**FIGURE 4:**
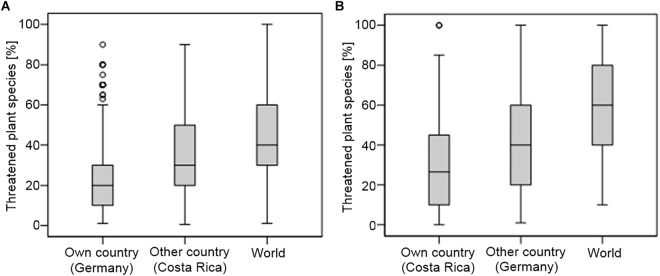
Boxplots showing the estimated percentages of threatened plant species in Germany, Costa Rica and worldwide by student biology teachers from Germany **(A)** and Costa Rica **(B)**.

Our results indicate that student science teachers have widely inaccurate ideas of the percentage of threatened plant species, when compared to scientific data. As hypothesized, students from both countries clearly showed a “spatial optimism bias” and an “overestimation bias” when estimating the percentage of threatened plant species in Germany, Costa Rica, and worldwide (Figure 4).

According to the current RL in Germany there are 0.5% of threatened plant species, in Costa Rica 1.0% and worldwide 4.5%, respectively (IUCN, 2011). Students from both countries largely overestimated^3^ the percentage of threatened plant species on the national (Germany: 40 times and Costa Rica: 30 times), transnational (Germany: 30 times and Costa Rica: 80 times), and on the global level (Germany: 9 times and Costa Rica: 14 times). Additionally, the estimated percentage of threatened plant species increased with greater distance from the students’ home country (Figure 4). Particularly for Germany the number of threatened plant species was strongly overestimated by both samples. German students estimated the percentage of threatened plant species more accurately than Costa Rican students for Germany (*F*_1,932_ = 154.28, *p* < 0.001) and worldwide (*F*_1,928_ = 96.38, *p* < 0.001). No difference was found in the accuracy of both samples estimates regarding the percentage of threatened plant species in Costa Rica (*F*_1,927_ = 2.49, *p* = 0.115). A detailed overview of the estimated percentages of threatened plant species in Germany, Costa Rica and globally can be found in Table 5.

**Table 5:** Estimated percentages of threatened plant species in Germany, Costa Rica, and worldwide by student biology teachers from Germany (D) and Costa Rica (CR) as compared to scientific data.

	Germany		Costa Rica		World	
Sample from	D	CR	D	CR	D	CR
Minimum	1	1	1	0	1	10
Maximum	90	100	90	100	100	100
Percentile 25	10	20	20	15	30	41
Median	20	40	30	30	40	60
Percentile 75	30	60	50	48	60	80
Actual percentage^∗^	0.5		1.0		4.3	
Overestimation^∗∗^	40.0	80.0	30.0	30.0	9.3	14.0

*All data in percent of total plant species of a country that are under threat [%]. ^∗^Threatened plant species counts taken from IUCN (2011): Germany = 0.5% (14 sp.), Costa Rica = 1.0% (116 sp.), worldwide = 4.3% (11,690 sp.). ^∗∗^Overestimation = median of estimate/actual percentage.*

## Discussion and Educational Implications

The main aim of the present study was to assess student teachers’ worldviews on the global distribution and loss of biodiversity. Two research questions guided our study: (1) To what degree do biology teachers from an industrialized country and a biodiversity hotspot share a common view on Earth’s biodiversity? (2) And in what ways do they differ? To address our research questions, we investigated the mental maps on the distribution and threat of global plant diversity and the perception of threatened plant species on the national, transnational and global levels of prospective biology teachers from Germany and Costa Rica.

### Student Teachers’ Mental Maps of High and Threatened Global Biodiversity

The most conspicuous common feature of the two subsamples’ composite mental maps is a phenomenon which we summarize under the term “brazilisation” of global biodiversity. More than half of our participants from both countries first and foremost associated Brazil with a country of a particularly high or threatened plant diversity. In scientific terms, Brazil is in 1st place regarding the world’s plant species richness (Groombridge and Jenkins, 2002) and in 5th place in terms of threatened plant species (IUCN, 2011). Thus, at first glance, our results seem very encouraging, as student teachers’ perception of Brazil’s biodiversity match quite well with the scientific view. However, we assume that this strong focus on Brazil might rather be based on lay people’s associations than on a deep scientific understanding. In the following we will try to justify this critical assumption.

The Brazilian Amazon region represents more than 40% of the total tropical rainforests area of the world and Brazil alone accounts for about 30% of the loss of the world’s tropical rainforests (Primack, 2010). The Earth Summit in Río de Janeiro in 1992 – and in particular the Amazon region in Brazil – gained a high media presence worldwide mainly focusing on the destruction of tropical rainforests and mass extinction of species (Väliverronen, 1998). Thus, the destruction of tropical rainforest has become a synonym for the rapid loss of species (Primack, 2010) and the latter has become a surrogate for global biodiversity loss (Haila and Kouki, 1994; Novacek, 2008). If we consider that children (Ballouard et al., 2011), the general public (Lindemann-Matthies and Bose, 2008) and even teachers (Cross, 1998; Michail et al., 2007) use the mass media as their major environmental information source, it is, thus, not surprising that prospective biology teachers’ worldviews of biodiversity are biased toward Brazil. This is further supported by recent research in environmental psychology showing that adolescents’ media exposure is closely associated with their biospheric value orientation and their environmental worldviews (Lee, 2011). In line with Michail et al. (2007) and Lee (2011) we assume that if teachers’ worldviews about global biodiversity issues are almost exclusively shaped through the mass media, it seems very likely that they are mainly based on a lay than on a scientific understanding. In this context, it is also worth considering that in-service science teachers are possibly even more dependent on the media as a source of information, because they have not come to know biodiversity in their tertiary education (see Michail et al., 2007).

Geographical research already showed that world regions that we do not know very well seem to be represented in our mental maps by so called “landmark countries” (Saarinen and MacCabe, 1995; Pinheiro, 1998; Saarinen, 2005). For example, the geographically complex South West Pacific region is commonly represented by Australia “as the isolated ‘church tower’ of that region” (Pinheiro, 1998; p. 335). We assume that Brazil, within the mental maps of prospective biology teachers from both countries, might not only stand as a representative for the high and threatened biodiversity of the South American region, but rather functions as the “landmark country” for global biodiversity *per se*.

From an educational point of view this intuitive focus on Brazil – may it be based on lay or scientific knowledge – has its drawbacks. A strong and intuitive focus on one particular country of the world might hinder student teachers to develop a global perspective on the distribution and loss of biodiversity, which is considered an integral part of ESD (e.g., Scott and Gough, 2004; Bolscho and Hauenschild, 2006; Scheunpflug and Asbrand, 2006; Menzel and Bögeholz, 2008) as well as a formal requirement of the national secondary science curriculums of both countries (Ministerio de Educación Pública [MEP], 2003, 2005; Sekretariat der Ständigen Konferenz der Kultusminister der Länder in der Bundesrepublik Deutschland [KMK], 2004). Student teachers’ worldviews and concepts – including their strong focus on Brazil – may be reflected in their future teaching practice (Cochran and Jones, 2003). In environmental education research it has already been shown that focusing too strong on selected flagship species such as the giant panda or the polar bear as biodiversity conservation tools to rise conservation awareness “detracts conservation efforts from other species and projects” (Ballouard et al., 2011; p. 6). Therefore, many authors demand that ESD “should encompass a wide diversity of species, notably by including less popular and neglected taxa” (p. 1) to develop positive attitudes toward global biodiversity (Ballouard et al., 2011; see also Balmford et al., 2002; Lindemann-Matthies, 2006). In the wake of this reasoning, we think that a mere restriction on the high and threatened biodiversity of a “flagship country” such as Brazil may not be the right way to promote a truly global perspective of biodiversity issues in pupils around the world.

Another common feature of the mental maps regarding threatened biodiversity is the predominant mentioning of industrialized countries such as United States, China, and Germany. From a scientific point of view, biodiversity is threatened most in tropical developing countries (Primack, 2010) and only China may be considered an industrialized country with a particularly threatened biodiversity (Mittermeier et al., 2004). This misconception of a particularly threatened biodiversity in industrialized countries is problematic as in most of the industrialized countries the loss of biodiversity is less urgent on a local scale than in developing countries (Mittermeier et al., 2004; Primack, 2010). Furthermore, based on this misconception, student teachers may conclude that industrialized countries make small efforts for biodiversity conservation and environmental protection. Thus, tertiary education programs should seize that industrialized countries also hold many species and ecosystems worth protecting, and that much of the biodiversity is actually protected. A concrete example to illustrate the biodiversity conservation efforts of industrialized countries in Europe may be the thematization of the European Habitats Directive on the conservation of natural habitats and of wild fauna and flora which forms the cornerstone of Europe’s nature conservation policy.

Interestingly, similar misconceptions of a particularly low biodiversity in industrialized countries have been found in a sample of Chilean and German high school students (Menzel and Bögeholz, 2008). They showed a so-called “lack of space-concept,” meaning that industrialized countries were thought to have extremely low biodiversity because of cramped cities and industries leaving no living space for animals and plants. In our sample this image might be so prominent that pre-service teachers even go a step further to ascribe industrialized countries a threatened biodiversity; possibly as a form of progression or superlative of an extremely low biodiversity. The meaning of and the differences between “extremely low” and “threatened” biodiversity seem to be mutually interdependent and even interchangeable. This equation of “extremely low” and “threatened” biodiversity might have merit in some cases, e.g., rare endemic island species (see Primack, 2010). However, at the country level this connection is not necessarily tenable. If a certain country holds a relatively small number of different species, this must not necessarily mean that these species are also threatened. Instead, it is even likely to be that many of those species will have a high number of individuals (Groombridge and Jenkins, 2002; Primack, 2010). We suggest that the just described misconception is essentially based on two fallacies: (1) a confusion of species richness and species abundance and (2) an insufficient differentiation between the different geographical scales of species diversity. Assuming that students confuse the low “number of different species in a given area” (= species richness) with a low “number of different individuals of a particular species in a given area” (= species abundance), it is reasonable that a low species diversity at the same time implicates its high threat. In general, species richness is differentiated along a gradient of the geographical scale of investigation from the habitat level (α-diversity), over the landscape level (γ-diversity) up to whole bio-geographical provinces (𝜀-diversity) (Magurran, 2004). We assume that student teachers in our sample possibly did not differentiate between these spatial levels. For example, if one assumes that the 𝜀-diversity of a certain species is low, this does not necessarily mean that its species richness at the country level or below is also low. Teachers need a clear understanding of the similarities and differences between “species richness” and “species abundance” as they are the most widely used measures of species diversity (Magurran, 2004; Primack, 2010). A discussion and comparison of “common species” and “rare species” might promote a better understanding of both concepts: In general, rare species are characterized in that they have (1) small geographical ranges, (2) small population sizes, and (3) specialized habitat requirements (Harrison et al., 2008). Common species, such as the Dandelion (*Taraxacum officinale*), have broad geographical ranges, large populations and less specialized habitat requirements, and, thus, are less susceptible to extinctions than rare species are (Harrison et al., 2008; Primack, 2010). Nevertheless, a rare species may also have huge population sizes in only a very limited geographical range or a highly specialized habitat. For example, the Common Glasswort (*Salicornia europaea*) or the Sea Aster (*Aster tripolium*), both are halophytic plants of northern Europe that are found only in salt marshes and estuaries, yet within these habitats, both plants are quite common.

Apart from the above-mentioned similarities, a number of ethnocentric distortions merged between Costa Rican and German student teachers’ composite mental maps. The majority of Costa Rican students almost exclusively focused on Latin American countries, especially on Costa Rica and adjacent countries, whereas other regions of the world such as Africa and Southeast Asia were largely marginalized. In contrast, German students’ notions were distributed more evenly across the globe including many European countries – albeit with relatively low percentages.

The inclusion of many Latin American countries in the Costa Rican sample and the inclusion of many European countries in the German sample may be explained by the “factor of proximity” and “cultural factors” as coined by Saarinen (2005). He found that students sketch maps of the world – and thus their mental maps – are more likely to include countries that are immediately adjacent (= factor of proximity) and countries which are culturally similar or closer to one’s home country (= cultural factor) (e.g., in terms of language, religion, and economy). It appears that these two factors identified by Saarinen (2005) also have an influence on our student teachers’ mental maps of global biodiversity. For example, Costa Rican students completely ignored the Asian region, which might be related to both, the large geographical distance and cultural differences. In addition, students from both countries largely neglected Africa, which as well might be based on cultural differences. To achieve a purely scientific view of global biodiversity, tertiary teacher education programs should critically discuss the possible distortive influences of proximity and cultural factors on student teachers’ biogeographic worldviews. Taking a psychological view, the distortions of the mental maps could also be interpreted in terms of the availability and representativeness heuristics (Kahneman and Tversky, 1972; Tversky and Kahneman, 1973), according to which individuals might mention some information more frequently because it is more readily *available* (the ease with which certain details can be brought to mind) and more *representative* in memory (people use categories to make estimates). Consistently, information about biodiversity might be more available and easier to categorize when it concerns one’s own country and the surrounding countries.

To sum up, our study showed that German students have a more global perspective on biodiversity and its loss, whereas Costa Ricans students have a more localized view with a special focus on Latin American countries. This is in line with previous research indicating that people from developing countries are more concerned about local environmental problems, whereas people from industrialized countries are often more focused on global environmental problems (Dunlap and Mertig, 1995; Holl et al., 1999). We assume that this difference may also be related to differences in geographic literacy between Germans and Costa Ricans. We assume that Costa Rican student teachers are less geographic literate than German students (see RoperASW, 2002), and, therefore, share a more localized biogeographic worldview. We argue that proceeding from their more local view on biodiversity, Costa Rican teacher training should include more global biodiversity issues, whereas German student teacher education should include more aspects of local biodiversity. As a starting point for participants in both countries, teacher educators may build up on German and Costa Rica students’ perception of a particularly threatened biodiversity in their respective country. A central theme for discussion could also be the many differences regarding the causes and structures of threats affecting the local biodiversity between industrialized and developing countries (see Primack, 2010). The local biodiversity in Germany is threatened for other reasons than in Costa Rica. Consequently, it should also be discussed that industrialized and developing countries are faced with different challenges in terms of biodiversity conservation.

Apart from the above mentioned we found that most students from both countries hold a naive biodiversity hotspot concept: One in four German participants and one in five Costa Rican participants assumed Brazil to have a particularly high and threatened biodiversity. In addition, thereto, one in six Costa Ricans sees his home country as a biodiversity hotspot. Other countries are considered as biodiversity hotspots only by single persons. Thus, our assumption, that Costa Ricans will be more likely to hold a biodiversity hotspot concept is only partially confirmed. However, we suggest that this naive biodiversity hotspot concept provides a good starting point for integrating global biodiversity conservation issues into tertiary teacher education programs. Further, we assume that biodiversity hotspots as a teaching topic offer great learning opportunities to reflect on socio-economic, as well as on ecological considerations for conserving global biodiversity. As most of our participants mainly considered tropical regions as areas with high and at the same time threatened biodiversity, teacher education programs should consider that biodiversity hotspots also occur outside tropical regions such as the Mediterranean regions and even in temperate countries such as Japan or New Zealand (Mittermeier et al., 2004).

### Perceived Threat of National, Transnational, and Global Biodiversity

In order to gain a deeper insight into student teachers’ worldviews on global biodiversity we explored whether student teachers would show an overestimation bias and a spatial optimism bias concerning their perception of threatened plant species diversity on the national, transnational and global levels.

#### Overestimation Bias

According to the latest RL in Germany 0.5% of the plant species are threatened, in Costa Rica 1% and 4.3% worldwide, respectively (IUCN, 2011). As hypothesized, student teachers from both countries drastically overestimated the percentage of threatened plant species on all spatial levels, when compared to scientific data. In this our results are in line with those obtained by others scholars, who found that the general public tend to overestimate the overall number of national and global plant species (Lindemann-Matthies and Bose, 2008) as well as the total number of species on earth and species extinctions per year (Dunning, 1997). It is often argued that people have problems in comprehending the magnitude of large numbers and that “beyond a certain level, numbers become abstract and unrelated to our everyday experiences” (Gehrt, 1996, p. 900). In the present study we have deliberately avoided to assess large numbers and restricted the possible range of numbers from 0 to 100 by asking for percentages of threatened plants. Nevertheless, our results showed that even assessed percentages of threatened plant species are consistently overestimated. This might be due to the fact that an estimate of the percentage of threatened plant species entails a cognitive handling of large numbers such as the total number of plants and the total number of threatened plants, which have to be weighed against each other. An indicator that the task might have required a high level of cognitive performance – at least for the Costa Rican students – is the great number of students who did not answer the question: 42.2% of the Costa Rican students and 10.3% of the German participants. Lindemann-Matthies and Bose (2008) argue that peoples’ responses to the ongoing loss of biodiversity and their support for conservation measures heavily depend on their conceptions of the numbers of species present and the ones being threatened or going extinct. In order to promote conservation attitudes and intentions among pupils and the wider community, quantitative data of certain species groups and their threat status have to be taught effectively in science classrooms. We think that this difficult task may not be achieved unless teachers are fully aware of the numerical scales involved, including the total numbers as well as the percentages of threatened species of certain taxonomic groups on the national and global level. In this context, Dunning (1997) already gave some practical suggestions on how to make large number relevant to students (e.g., through the use of more graphical conceptualizations of the dimensions of biodiversity loss). Some authors have already shown that the current global human population and its annual growth are also drastically overestimated by most people (Meffe, 1994; Dunning, 1997). As human overpopulation is one of the greatest threats to biodiversity (Millennium Ecosystem Assessment [MEA], 2005), we follow Gehrt’s (1996) suggestion that biodiversity relevance should always accompany figures of human population growth and vice versa.

#### Spatial Optimism Bias

In our study student biology teachers from both countries showed a strong “spatial optimism bias,” meaning that the assumed percentage of threatened plant species increased from the national, over the transnational up to the global spatial level. In this study, we demonstrated for the first time that the “spatial optimism bias” applies to concrete transnational assessments. Thus, we could show that the “spatial optimism bias” not only occurs when assessing environmental problems on different geographical scales (e.g., from the local to the global) but also in concrete situation when it comes to evaluate environmental problems in one’s home country in comparison to another country. Our results suggest that in most cases one’s home country will be evaluated better concerning its status of biodiversity then those of other countries, no matter if one lives in a biodiversity hotspot or an industrialized country. From a global perspective, this extended view of the spatial optimism bias has immense educational shortfalls, for example if the majority of science teachers around the world may think that their local biodiversity is in good conditions in comparison to that of other countries, they might not feel a special need to address local biodiversity issues in classroom and outdoor activities. However, first hand nature experiences with local flora and fauna are seen as a crucial prerequisite for the development of environmental knowledge and values in pupils (Bögeholz, 2006).

However, it should be considered that the students were first asked about their own country, followed by the other country in the study, before they were finally asked about the percentage of threatened plant species globally. A different arrangement of the items might have led to different results, even if this can be assumed to be relatively unlikely (cf. Dunning, 1997; Lindemann-Matthies and Bose, 2008). Nevertheless, a randomization of the items in the questionnaire would certainly have been a viable alternative. Due to the special sample used, it should be noted that the results of the present study cannot be easily transferred for use in studies pertaining to students in other fields of study, or the general population.

Furthermore, it should be noted that we did not consider in this study whether socio-economic features of both student teacher samples (e.g., housing, family incomes, types of employment during the university course) could have an impact on their mental maps of global biodiversity. This question in relation to the socio-economic status of the students may be deeply approached in future studies.

## Ethics Statement

The study was carried out in accordance with the Declaration of Helsinki and APA’s Ethical Principles of Psychologists and Code of Conduct (American Psychological Association, 2016; Nijhawan et al., 2013). Anonymity was guaranteed and the participation was on a voluntary basis. Thus, all participants had the chance to decline to participate and to withdraw from the research at any time. Informed written consent of the participants was implied through survey completion. Additionally, all participants were introduced to the aim of the study. As our investigation was conducted by an anonymous questionnaire in an educational setting in the presence of the respective university professor, our research involved no risk to our subjects. Moreover, our research will not adversely affect the rights and welfare of our subjects, had no medical background and assessed no sensitive personal data. In consequence, an ethics approval was not required as per institutional (Universität Osnabrück, 2017) and national guidelines (German Research Foundation, DFG, 2018).

## Author Contributions

Both authors made substantial contributions to the conception and design of the work as well as for the analysis and interpretation of data.

## Conflict of Interest Statement

The authors declare that the research was conducted in the absence of any commercial or financial relationships that could be construed as a potential conflict of interest.

## Footnotes

1 https://grass.osgeo.org

2 https://qgis.org/de

3 Overestimation = median of estimates/actual percentage
